# Antifungal Activity of Ocimum tenuiflorum and Ocimum gratissimum Herbal Formulation-Based Oral Rinse Against Candida albicans

**DOI:** 10.7759/cureus.67111

**Published:** 2024-08-18

**Authors:** Nehal Safiya S, Remmiya Mary Varghese, Aravind Kumar S, Rajeshkumar Shanmugam

**Affiliations:** 1 Dentistry, Saveetha Dental College and Hospitals, Saveetha Institute of Medical and Technical Sciences, Saveetha University, Chennai, IND; 2 Orthodontics and Dentofacial Orthopaedics, Saveetha Dental College and Hospitals, Saveetha Institute of Medical and Technical Sciences, Saveetha University, Chennai, IND; 3 Nanobiomedicine Lab, Centre for Global Health Research, Saveetha Medical College and Hospital, Saveetha Institute of Medical and Technical Sciences, Chennai, IND

**Keywords:** candida albicans, antifungal activity, herbal oral rinse, ocimum gratissimum, ocimum tenuiflorum

## Abstract

Background

Candida albicans is a common fungal pathogen responsible for oral infections, posing significant health challenges. Traditional antifungal treatments often come with side effects and resistance issues, highlighting the need for effective natural alternatives. O. tenuiflorum and Ocimum gratissimum are known for their medicinal properties, including antifungal activity.

Objective

This study aimed to evaluate the antifungal effectiveness of an O. tenuiflorum and O. gratissimum herbal formulation-based oral rinse against C. albicans.

Methods

Antifungal activity was measured using agar well diffusion, time-kill curve assays, and analyses of cytoplasmic and protein leakage. The herbal rinse was tested at concentrations of 25 µg/mL, 50 µg/mL, and 100 µg/mL, and compared to a commercial oral rinse.

Results

The herbal rinse demonstrated strong antifungal effects that increased with concentration. At 100 µg/mL, it produced a 13 mm zone of inhibition, outperforming the commercial rinse's 11 mm. The time-kill assay revealed that the 100 µg/mL concentration reduced fungal counts to 103 CFU/mL within 5 hours, on par with the commercial rinse. Cytoplasmic leakage analysis showed an optical density of 0.42 at 100 µg/mL, close to the commercial rinse's 0.45. Protein leakage analysis indicated an optical density of 0.52 at 100 µg/mL, slightly higher than the commercial rinse's 0.51.

Conclusion

The O. tenuiflorum and O. gratissimum herbal formulation-based oral rinse exhibit potent antifungal activity against C. albicans, rivaling and even surpassing commercial rinses at higher concentrations. This study underscores the potential of this natural oral rinse as a powerful alternative for managing oral fungal infections, meriting further research and clinical trials to confirm its long-term safety and efficacy.

## Introduction

Candida albicans significantly contributes to oral infections, particularly in immunocompromised individuals. It colonizes the oral cavity, forming resilient biofilms on mucosal surfaces and dental materials [1]. Its virulence factors, including adherence, phenotype switching, and immune interference, enhance pathogenicity [2]. Host factors like xerostomia, smoking, oral prostheses, dental caries, diabetes, and cancer treatment facilitate infection development. C. albicans often coexists with bacteria like Streptococcus mutans, enhancing biofilm virulence [3]. Oral candidiasis manifests in various forms, causing mucosal inflammation. Biofilm formation involves adhesion, polysaccharide matrix production, environmental adaptation, and quorum sensing. Prevention involves addressing host factors and using antimicrobial oral hygiene products to reduce Candida levels and virulence [4,5].

Traditional oral care products for treating C. albicans infections face several challenges. These include antifungal resistance due to overuse, significant side effects from synthetic drugs, and a limited spectrum of activity that may not cover all Candida strains [6]. Maintaining therapeutic concentrations in the oral cavity is difficult because of constant saliva flow, which leads to subtherapeutic levels. Patient compliance is also an issue, especially with long-term use of products like chlorhexidine gluconate mouth rinses, which can have side effects [7,8]. Additionally, the high cost and limited accessibility of synthetic antifungal drugs pose problems, particularly in resource-limited settings. While herbal and natural products are proposed as alternatives due to their lower cost and fewer side effects, more research is needed to confirm their efficacy and safety [9, 10].

Herbal oral care products have emerged as a promising alternative to synthetic antifungal medications for treating C. albicans infections [11]. They generally exhibit lower toxicity and fewer side effects due to their natural origins and complex compositions. Many herbs, such as garlic, ginger, grapefruit seed extract, oregano, and berberine, have potent antimicrobial and antifungal properties [12,13]. Additionally, certain herbal medicines can enhance the innate immune response by regulating pattern recognition receptors and activating immune cells, aiding the body in fighting infections more effectively. Herbal remedies are also less likely to induce resistance compared to single-compound synthetic drugs because of their multiple active compounds [14]. Furthermore, they are often more accessible and cost-effective, especially in resource-limited settings. While these benefits highlight the potential of herbal oral care products in managing oral candidiasis, further research is necessary to confirm their efficacy and safety in clinical applications [15].

In this present study, a herbal formulation was prepared using extracts from Ocimum tenuiflorum and Ocimum gratissimum leaves. This formulation was then used to create an oral rinse, which was tested for its antifungal activity against C. albicans using the agar-well diffusion technique, time-kill curve assay, cytoplasmic leakage, and protein leakage analysis.

## Materials and methods

Preparation of O. tenuiflorum and O. gratissimum herbal formulation

Fresh leaves of O. tenuiflorum and O. gratissimum were collected and thoroughly washed with distilled water to remove any surface contaminants. The leaves were then shade-dried at room temperature until completely dehydrated. Once dried, the leaves were finely powdered using a mechanical grinder. A solution was prepared by combining 1 g of each powdered leaf with 100 mL of distilled water. This mixture was heated at 60 °C for 15-20 minutes using a heating mantle. After boiling, the mixture was gradually filtered using filter paper.

Preparation of O. tenuiflorum and O. gratissimum herbal formulation-based oral rinse

**Figure 1:**
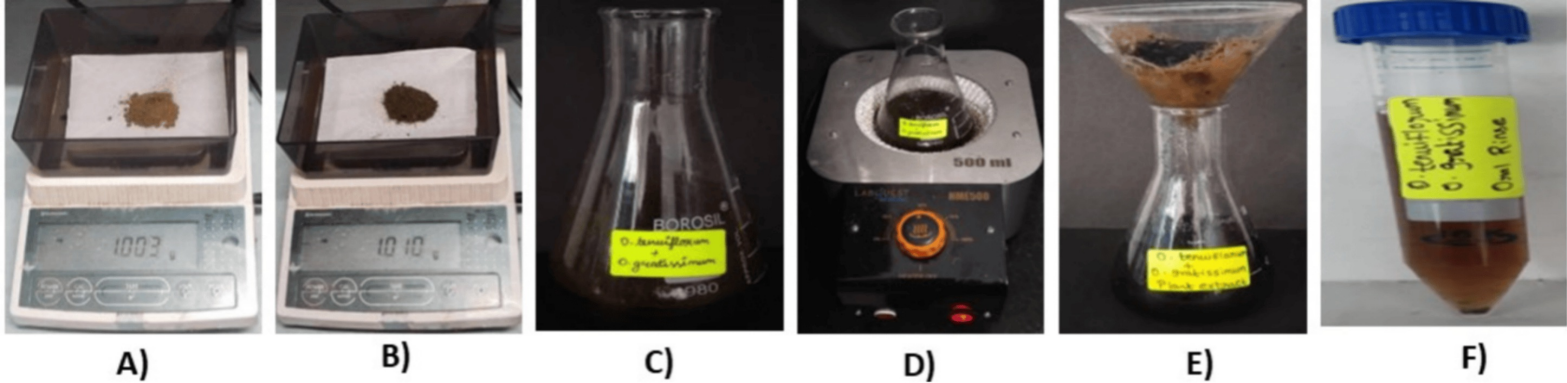
Preparation of herbal formulation-based oral rinse: (A) Ocimum tenuiflorum powder; (B) Ocimum grattissimum powder; (C) addition of both powders in 100 g distilled water; (D) boiled using a heating mantle; (E) filtered herbal formulation; (F) Ocimum tenuiflorum and Ocimum grattissimum-based oral rinse.

In Figure 1, the O. tenuiflorum and O. gratissimum herbal formulation-based oral rinse were prepared by combining 0.3 g of sucrose, 0.1 g of sodium lauryl sulfate, 0.001 g of sodium benzoate, and 500 µL of herbal formulation in 10 mL of distilled water.

The sucrose acted as a sweetener, sodium lauryl sulfate as a foaming agent, and sodium benzoate as a preservative. The mixture was thoroughly mixed to create a herbal formulation-based oral rinse.

Antifungal activity: Agar well diffusion technique

The antifungal activity of the O. tenuiflorum and O. gratissimum herbal formulation-based oral rinse was evaluated using the agar well diffusion technique against C. albicans. A standardized inoculum of C. albicans (approximately 106 CFU/mL) was evenly spread onto the surface of sterile Rose Bengal agar plates. Wells of 9 mm diameter were punched into the agar using a sterile cork borer, and 100 µL of the herbal formulation-based oral rinse at concentrations of 25, 50, and 100 µg/mL were added into the respective wells. A commercial oral rinse was used as a standard for comparison. The plates were incubated at 37 °C for 24 hours, and the zones of inhibition around the wells were measured in millimeters.

Protein leakage analysis

Protein leakage from C. albicans cells was assessed to determine the antifungal efficacy of the herbal formulation-based oral rinse. The cells were treated with the herbal formulation-based oral rinse at concentrations of 25, 50, and 100 µg/mL, as well as a commercial oral rinse (standard) and untreated control. After treatment, the samples were centrifuged, and the supernatant was collected. The protein content in the supernatant was quantified by measuring the optical density at 280 nm using a UV-Vis spectrophotometer, indicating the extent of protein leakage from the cells.

Cytoplasmic leakage analysis

The cytoplasmic leakage was analyzed to evaluate the membrane-disruptive action of the herbal formulation-based oral rinse against C. albicans. The fungal pathogen was treated with the herbal formulation-based oral rinse at different concentrations (25, 50, and 100 µg/mL), along with a commercial oral rinse and an untreated control. After incubation, the treated samples were centrifuged, and the supernatant was collected. The optical density of the supernatant was measured at 260 nm using a UV-Vis spectrophotometer to determine the release of cytoplasmic contents, reflecting cell membrane damage.

Time-kill curve assay

The time-kill curve assay was conducted to evaluate the rate at which the herbal formulation killed C. albicans over five hours. An inoculum of C. albicans standardized to approximately 106 CFU/mL was treated with the herbal formulation-based oral rinse at concentrations of 25, 50, and 100 µg/mL, alongside a commercial oral rinse (standard) and untreated control. Aliquots were taken at specific time intervals (0, 1, 2, 3, 4, and 5 hours), serially diluted, and their optical density was measured using an ELISA plate reader (BioTek Instruments, Winooski, VT) at 600 nm. This method allowed for the plotting of time-kill curves to compare the effectiveness of the herbal formulation concentrations, the commercial oral rinse, and the untreated control in reducing C. albicans over time.

## Results

Antifungal activity using agar well diffusion technique

**Figure 2:**
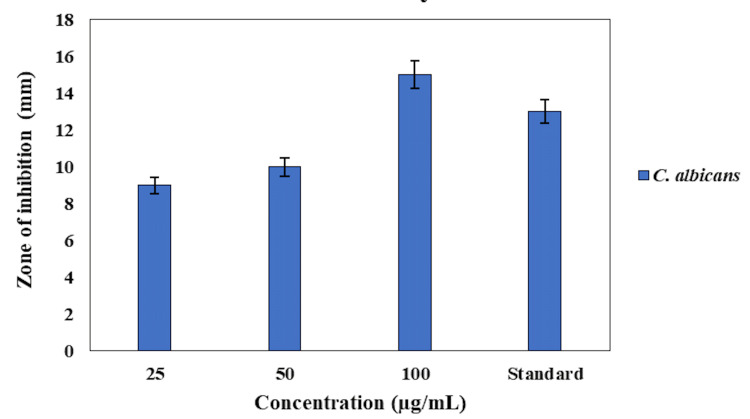
Antifungal activity of herbal formulation-based oral rinse against Candida albicans compared with a standard commercial oral rinse.

In Figure 2, the antifungal activity of the O. tenuiflorum and O. grattissimum herbal formulation-based oral rinse against C. albicans was assessed by measuring the zone of inhibition in mm at concentrations of 25, 50, and 100 µg/mL, compared with a commercial oral rinse as a standard. The herbal formulation at 25 µg/mL exhibited a moderate antifungal effect with a zone of inhibition measuring approximately 9 mm. Increasing the concentration to 50 µg/mL resulted in an expanded zone of inhibition of around 10 mm. The highest concentration of 100 µg/mL demonstrated the most significant antifungal activity, with a zone of inhibition measuring approximately 13 mm. In comparison, the commercial oral rinse showed a zone of inhibition of 11 mm. These results indicate that the O. tenuiflorum and O. grattissimum herbal formulation-based oral rinse exhibit effective antifungal activity against C. albicans, with the highest concentration outperforming the commercial standard. The results suggest that this herbal formulation could serve as a potent natural alternative for antifungal oral care, providing substantial inhibition of C. albicans growth.

Time-kill curve assay

**Figure 3:**
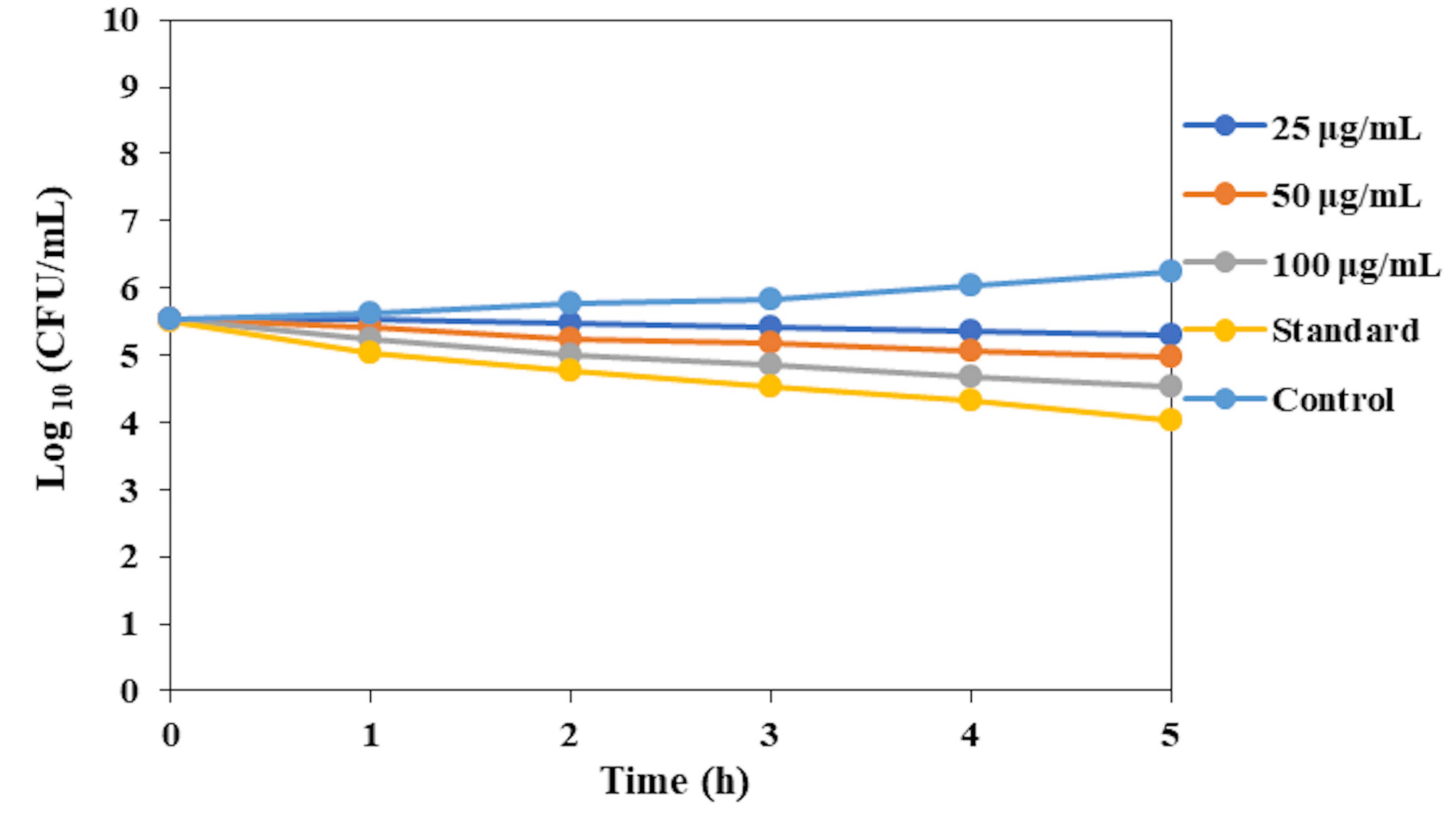
Time-kill curve assay of herbal formulation-based oral rinse against Candida albicans.

In Figure 3, the time-kill curve assay for the O. tenuiflorum and O. grattissimum herbal formulation-based oral rinse was conducted to evaluate its antifungal efficacy against C. albicans over five hours at concentrations of 25, 50, and 100 µg/mL, compared with a commercial oral rinse (standard) and an untreated control. The initial fungal count was approximately 106 CFU/mL. The control group showed no significant decline in fungal counts, maintaining stability throughout the five hours. At the 25 µg/mL concentration, the herbal formulation exhibited a gradual decrease in fungal counts, reaching approximately 105 CFU/mL by the fifth hour. The 50 µg/mL concentration demonstrated enhanced antifungal activity, reducing the fungal counts more effectively to around 104 CFU/mL within the same timeframe. The highest concentration of 100 µg/mL showed the most significant reduction, with fungal counts decreasing to approximately 103 CFU/mL by the fifth hour. The commercial oral rinse (standard) exhibited a similar trend to the 100 µg/mL concentration of the herbal formulation, with fungal counts also reducing to approximately 103 CFU/mL. These results indicate that the O. tenuiflorum and O. grattissimum herbal formulation-based oral rinse has a strong antifungal effect against C. albicans, with its efficacy being concentration-dependent. The highest concentration tested (100 µg/mL) demonstrated comparable activity to the commercial standard, indicating its potent antifungal properties.

Cytoplasmic leakage analysis

**Figure 4:**
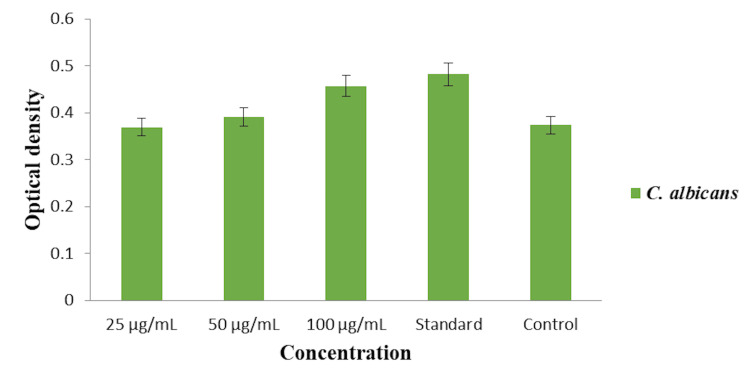
Cytoplasmic leakage analysis of herbal formulation-based oral rinse against Candida albicans.

In Figure 4, the cytoplasmic leakage analysis was conducted to evaluate the antifungal efficacy of the O. tenuiflorum and O. grattissimum herbal formulation-based oral rinse against C. albicans by measuring the optical density at different concentrations (25, 50, and 100 µg/mL) and comparing it to a commercial oral rinse (standard) and an untreated control. At the 25 µg/mL concentration, the herbal formulation exhibited an optical density of approximately 0.35, indicating a moderate level of cytoplasmic leakage. Increasing the concentration to 50 µg/mL resulted in an optical density of around 0.38, showing enhanced cytoplasmic leakage. The highest concentration of 100 µg/mL demonstrated significant cytoplasmic leakage with an optical density of approximately 0.42. The commercial oral rinse showed an optical density of about 0.45, indicating its effectiveness in causing cytoplasmic leakage in C. albicans. The untreated control group exhibited the lowest optical density of approximately 0.32, reflecting minimal cytoplasmic leakage. These results indicate that the O. tenuiflorum and O. grattissimum herbal formulation-based oral rinse demonstrate effective antifungal activity against C. albicans, with the highest concentration (100 µg/mL) resulting in significant cytoplasmic leakage, approaching the efficacy of the commercial oral rinse.

Protein leakage analysis

**Figure 5:**
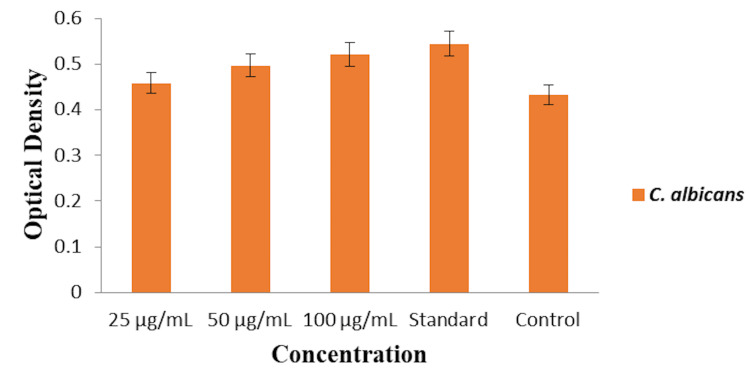
Protein leakage analysis of herbal formulation-based oral rinse against Candida albicans.

The protein leakage analysis was conducted to assess the antifungal efficacy of the O. tenuiflorum and O. grattissimum herbal formulation-based oral rinse against C. albicans by measuring the optical density at different concentrations (25, 50, and 100 µg/mL) and comparing it to a commercial oral rinse (standard) and an untreated control. At the 25 µg/mL concentration, the herbal formulation-based oral rinse exhibited an optical density of approximately 0.45, indicating a moderate level of protein leakage. Increasing the concentration to 50 µg/mL resulted in an optical density of about 0.48, showing an increase in protein leakage. The highest concentration of 100 µg/mL demonstrated a further increase in protein leakage, with an optical density of approximately 0.52. The commercial oral rinse showed an optical density of about 0.51, indicating its effectiveness in causing protein leakage in C. albicans. The untreated control group exhibited the lowest optical density of approximately 0.38, reflecting minimal protein leakage. These results indicate that the O. tenuiflorum and O. grattissimum herbal formulation-based oral rinse demonstrate effective antifungal activity against C. albicans, as shown in Figure 5, with the highest concentration (100 µg/mL) resulting in significant protein leakage, comparable to the commercial oral rinse.

## Discussion

The present study aimed to evaluate the antifungal activity of O. tenuiflorum and O. gratissimum herbal formulation-based oral rinses against C. albicans. The results demonstrated that this herbal formulation-based oral rinse exhibits significant antifungal properties, with its efficacy being concentration-dependent [16]. The herbal formulation-based oral rinse showed a progressive increase in the zone of inhibition against C. albicans with increasing concentrations. At 25 µg/mL, the zone of inhibition was approximately 9 mm, which expanded to 10 mm at 50 µg/mL and reached 13 mm at 100 µg/mL. In comparison, the commercial oral rinse exhibited a zone of inhibition of 11 mm at its standard concentration. This suggests that the highest concentration of the herbal formulation surpasses the commercial rinse in antifungal efficacy [17]. The time-kill curve assay revealed a concentration-dependent reduction in fungal counts. The 100 µg/mL concentration of the oral rinse reduced the fungal counts to approximately 103 CFU/mL within five hours, comparable to the commercial oral rinse. Lower concentrations (25 and 50 µg/mL) showed less pronounced but still significant reductions in fungal counts [18,19].

The herbal formulation-based oral rinse induced cytoplasmic leakage in C. albicans, with optical density measurements increasing with concentration. The highest concentration (100 µg/mL) resulted in significant cytoplasmic leakage, with an optical density of approximately 0.42, nearing the effectiveness of the commercial oral rinse (0.45). Similar to cytoplasmic leakage, protein leakage analysis showed that the herbal formulation-based oral rinse caused a concentration-dependent increase in optical density. At 100 µg/mL, the optical density was 0.52, slightly higher than the commercial oral rinse (0.51), indicating potent antifungal activity [20]. The findings of this study align with the existing literature on the antifungal properties of O. species. Manzoor et al. (2010) reported that O. sanctum essential oil exerts antifungal activity by disrupting ergosterol biosynthesis and membrane integrity, mechanisms that are likely shared by the O. tenuiflorum and O. gratissimum formulations used in this study [21].

Jayasankar et al. developed a herbal antifungal gel containing Origanum vulgare and Syzygium aromaticum essential oils, which showed significant antifungal activity against oral C. albicans. The comparable efficacy of the Ocimum-based formulation in this study highlights the potential of herbal formulations in oral healthcare [22]. Hsu et al. conducted a systematic review of herbal extracts with antifungal activity against C. albicans, identifying several plant-based compounds with significant antifungal properties. The current study adds to this body of knowledge by demonstrating the specific efficacy of O. tenuiflorum and O. gratissimum [23]. Studies by Yigit et al. and Adwan et al. evaluated the antifungal activity of toothpastes against oral Candida isolates, showing that conventional and herbal toothpastes can effectively inhibit fungal growth. The results of this study suggest that the Ocimum-based oral rinse could serve as a potent natural alternative to conventional oral care products [24,25].

Implications for future research

The promising results of this study indicate the need for further research to optimize the formulation and evaluate its long-term safety and efficacy. Clinical trials should be conducted to confirm these in vitro findings and assess the potential of this herbal formulation in preventing and treating oral fungal infections in a real-world setting. The O. tenuiflorum and O. gratissimum herbal formulation-based oral rinse exhibit strong antifungal activity against C. albicans, with its efficacy being comparable to, and in some cases surpassing, a commercial oral rinse. This study highlights the potential of herbal formulations as effective alternatives in oral healthcare, offering a natural solution for managing fungal infections.

Limitations

The in vitro nature of the study, bioavailability, metabolism, and systemic effects of the herbal formulation were not accounted for in this study. C. albicans might have different resistance mechanisms compared to other fungi. The results might not predict efficacy against strains with varying resistance profiles. The stability of the herbal rinse over time and under different storage conditions might affect its antifungal properties. This factor is not usually addressed in in vitro studies. These limitations underscore the need for further in vivo research to address these gaps and enhance the overall robustness and translational applicability of our findings.

## Conclusions

This study categorically demonstrates that the O. tenuiflorum and O. gratissimum herbal formulation-based oral rinses possess significant antifungal activity against C. albicans. The findings highlight a clear concentration-dependent efficacy, with the highest concentration surpassing the performance of a commercial oral rinse. The herbal formulation also induced notable cytoplasmic and protein leakage in the fungal cells, further confirming its potent antifungal properties. These results revealed the potential of the O. tenuiflorum and O. gratissimum herbal formulations as a natural and effective alternative to conventional antifungal treatments. This study supports the viability of herbal formulations in developing efficacious antifungal therapies, providing a promising option for the management of oral fungal infections.
